# Supplementary figures and images for: Specificity of spiders among fear- and disgust-eliciting arthropods: Spiders are special, but phobics not so much
Source: PLoS One. 2021 Sep 23;16(9):e0257726. doi: 10.1371/journal.pone.0257726 (PMC8460016; doi:10.1371/journal.pone.0257726)

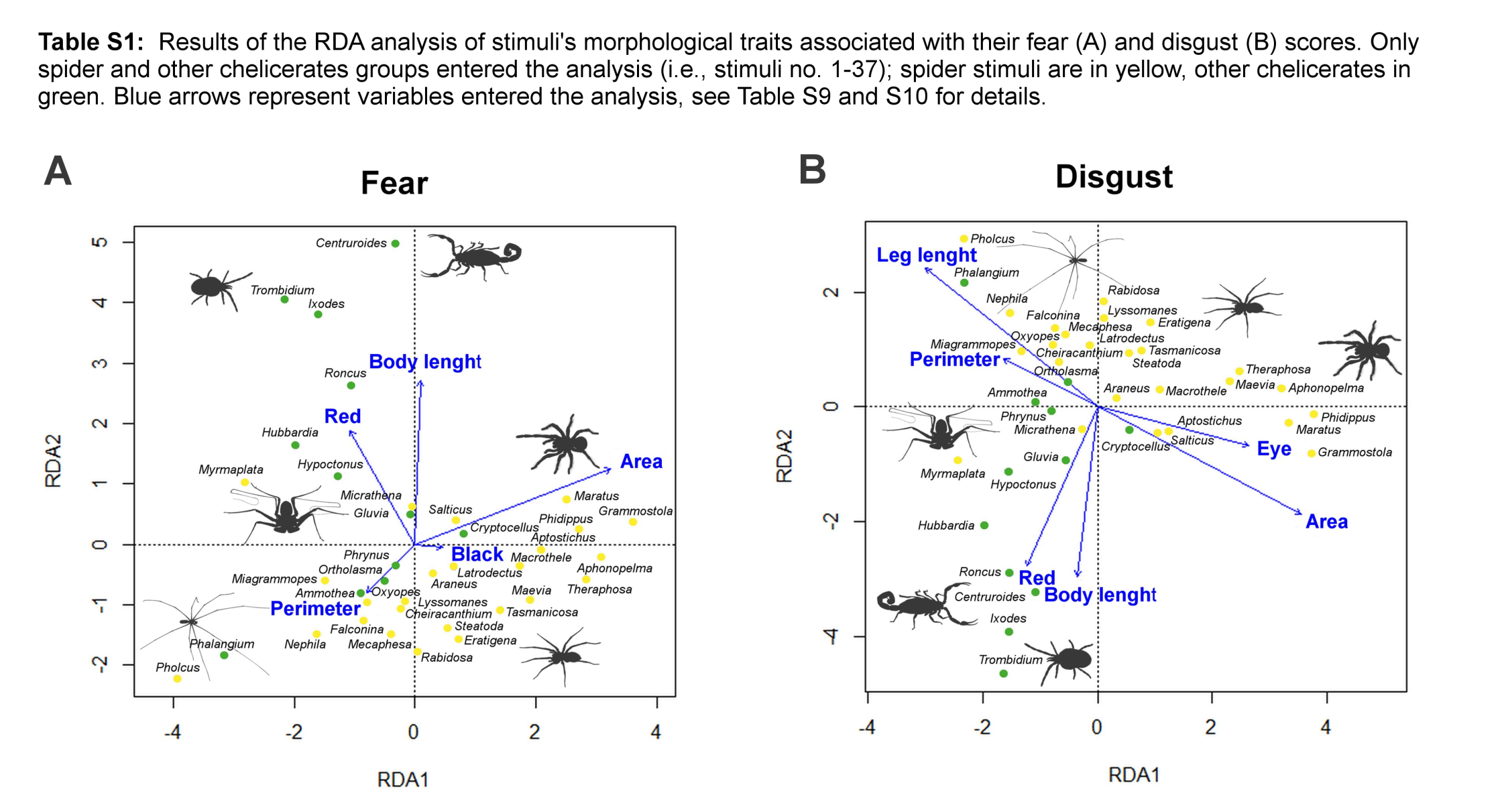

Supplement: S1 Fig — (TIF) [file pone.0257726.s001.tif]
