# Supplementary material for: Specificity of spiders among fear- and disgust-eliciting arthropods: Spiders are special, but phobics not so much
Source: PLoS One. 2021 Sep 23;16(9):e0257726. doi: 10.1371/journal.pone.0257726 (PMC8460016; doi:10.1371/journal.pone.0257726)
Supplement: Attachment — Submitted filename: Response_to_Reviewers.docx [file pone.0257726.s004.docx]

**Response to Reviewers**

We thank both reviewers for reading the manuscript and commenting on our work. Here we wrote the answers to all the questions and described the modifications we made in the manuscript based on the comments.

**Reviewer 1:** Unfortunately, I was not able to assess the statistical analyses in detail as I was unable to review the supplements. In the text it states an Excel file summarizing details on statistics, however, even after consulting the Editor, no such file was available to me. Some of my remarks may thus ground on false believes about your data structure on my part. Please provide the missing files and data, so that the review process can be continued with all information necessary.

*Line 41: grammar: "circuits are"*

**ACCEPTED:** The sentence has been corrected.

**GENERAL EXPLANATION to *Line 76, 77* and *83*:**

All the remaining questions (line 76, 77, 83) challenge the assumption of evolutionary roots of heightened emotions fear and disgust to spiders. We are closely familiar with paper of **Davey (1994)** who propose this idea: “ It has generally been accepted that fear of spiders is an example of a biologically prepared fear. The theory of biological preparedness assumes that most feared objects (such as spiders) are those which had been hazardous for our pretechnological ancestors (other hazardous stimuli would include snakes, darkness, heights, etc.). The selection pressures generated by these hazardous stimuli had then resulted in the evolution of phylogenetically-based predispositions to associate these pretechnologically "prepared" stimuli with traumatic consequences (Seligman, 1970, 1971). Since at least some spiders are lethally venomous, the implicit assumption is that this is the selection factor which generated a biological predisposition to associate spiders with fearful consequences.“

Latter was added arguments that supports evolutionary roots of existence of “prepared” stimuli like early visual attention, quick neural processing, and sometimes also quick learning (as wos originally proposed), strong psychophysiological emotional response, existence of attentional and emotional bias in children or cross-cultural agreement on attitude to the spiders. However, the evidence for each particular argument is complicated in spiders, not so clear like with snakes (see below). From all these arguments we agree with the reviewer, the existence of specific recognition of spiders in 5-month (Radkinson and Derringer 2008) or 6-month old (Hoehl et al., 2017) children is the strongest argument (see also below).

Why we thing that heightened fear and disgust associated with spiders in adults is one of the supportive arguments towards evolutionary roots. Actually, this is based on our experience with people suffering from specific phobia from spiders and snakes. We work with them several years, they have high and dysregulated fear and also disgust from spider stimuli. Interestingly, we ask them if they have any memory of initial accident with feared object, but we interview up to date more than 80 spider and snake phobic and they haven’t any such memory. Actually they have fear as long as they remember (usually they report phobic fear from the age of four years). Intensity of subjectively perceived phobic fear and disgust may change a little bit, but it is fully present very early in life. Even normal people when evaluate emotions caused by different animals that triggered animal phobia evaluate spiders highly according fear and disgust (however, some parasites have also very high, even higher emotional evaluation) comparing to other animal species including the snakes (Polak et al. 2020). We hypothesize, that high and specific fear and disgust evaluation shared by people with very different background may pointed out to the shared pre-prepared emotional category (may be, may be not evolutionary based). The cross-cultural study, should shed light on the topic.

We have two main points where we see the gap in original Davey’s statement. First, there is minimum spider species, that are dangerous to humans. Second, this evolutionary theory explains only evolution of fear, not disgust, which is highly associated with spiders. Both emotions are tightly connected with spiders and may be also with other arthropod species. In series of experiments (this is the first one) we try to find which species (group of species) should be potential model species for evolution of fear and disgust. However, both emotions have different evolutionary roots, fear is thought to be connected with reaction to the strong immediate threat, the disgust with delayed hidden threat like, contact with diseases, poisonous food, dirt… So the fear and disgust should be generalized from two completely different animal species (or wider group of species).

We hypothesise that even if the reaction to the spiders are somehow preprepared, the evidence is not so clear because it is transmitted (generalized) from other more relevant models.

As our research group contains psychologists as well as ethologists and people primarily focused on zoology. We found interesting the term “prepared” actually is very close to the classical meaning of ethological term innate – it means existing as fully complex recognitional and motorical pattern “pre-existing “ in mind of the animal (it doesn’t mean purely genetically based). When animal approach the proper stimulus, it appears suddenly as a complex behavioural pattern, which is shared by all the member of the species.

Here, we see the connection with categorization theory and evolutionary theory of emotions for fear and disgust. Categorization as a cognitive process presuppose the existence if specific category of the stimuli inside the mind. Moreover, emotional stimuli in general, and those containing emotionally and evolutionary relevant stimuli are thought to be categorized specifically. We thing, that if the people with very different experience and different level of fear of spiders share similar perceptual or emotional category (containing spiders and model species) it is supportive argument for “preprepared” stimulus category in the mind. However, there is still argument, that such categorization process should be formed via shared cultural influence during development. The developmental studies which currently appears brings argument for both preprepared recognition and emotional reaction, but also for potential of associative learning in infants early in life. Detail cross-cultural studies should shed new light on the topic.

Davey, G. C. (1994). The" disgusting" spider: The role of disease and illness in the perpetuation of fear of spiders. Society & Animals, 2(1), 17-25.

Hoehl, S., Hellmer, K., Johansson, M., & Gredebäck, G. (2017). Itsy bitsy spider…: Infants react with increased arousal to spiders and snakes. Frontiers in psychology, 8, 1710.

Polák, J., Rádlová, S., Janovcová, M., Flegr, J., Landová, E., & Frynta, D. (2020). Scary and nasty beasts: Self‐reported fear and disgust of common phobic animals. British Journal of Psychology, 111(2), 297-321.

Rakison DH, Derringer J. Do infants possess an evolved spider-detection mechanism?. 807 Cognition. 2008; 107(1), 381-393.

*Line 76:* *Please describe in more detail why this would point towards evolutionary roots of negative emotions. Participants investigated in the referenced studies were adults. Thus, one may argue that cultural, learned responses to spiders were the main source of these self-rate results rather than endophenotypes or genetic markers.*

**EXPLAINED**: The reaction to the spiders are very specific, present in very young age (6-month, 9 month childrens express it) and there are one study showing cross-cultural agreement on attitude towards the spider. Thus influence of cultural stereotype will not be the only explanation. Moreover, when people with very different social background, age and education shows agreement and categorize similarly the stimuli according the emotions it should be due to pre-existing perceptual category or very widely shared cultural bias. In our opinion, very detailed investigation of emotional evaluation and categorization of spiders and other invertebrates may shed light on the topic.

*Line 77: Please describe in detail why this qualifies as a line of evidence for evolutionary roots. From my point of view, the fact that both, fear and disgust are elicited, does not necessarily point towards biological preparedness.*

**EXPLAINED:** We view the high level of specific fear and disgust evoked by spiders.as a accompanying sign which is consistent with the theory about biological preparedness. However, it is only the supportive argument. The existence of pre-prepared category in the mind of very young children, or even in the mind of adult people with very different experience (the focus of this article) or future cross-cultural studies should be more supportive arguments. We soften the argumentation and change the order of arguments, we rewrite the paragraph. We know, that view of spiders as evolutionary pre-prepared stimuli is somewhat weird, but we try to explore the role of generalization with other more relevant stimuli (see above for general arguments).

For evolution of disgust, was proposed a little bit different evolutionary theory, however experimentally testable assumptions are not so clear like in the case of the fear module We suppose that high level of subjectively perceived disgust caused by spiders and other invertebrates may be consistent with the following theory:

It is supposed that with disgust another kind of danger has been lurking around, potentially far more significant, represented by parasites and bacterial or viral pathogens. However, as the nature of the threat posed by pathogens is qualitatively different from an imminent physical attack, fear could not be the right response anymore. Therefore, our ancestors as distant as early mammals have evolved another special mechanism known as the behavioural immune system with disgust as the key emotion. Disgust, compared to fear, utilizes a different neural network including the anterior insular cortex, basal ganglia, ventrolateral and medial prefrontal cortex, anterior temporal cortex, and visual cortex. Physiological changes are associated with activation of the parasympathetic nervous system leading to lower blood pressure and galvanic skin response (GSR). The behavioural pattern includes withdrawal, distancing from or dropping the disgusting object (Curtis, 2011).

However, if this high emotional evaluation of spiders according the disgust is caused by the shared cultural stereotype o rif it has evolutionary roots and generalization of some invertebrates conected with avoidance of desease transmittion is in play may resolve cross-cultural studies.

Curtis, V., De Barra, M., & Aunger, R. (2011). Disgust as an adaptive system for disease avoidance behaviour. Philosophical Transactions of the Royal Society B: Biological Sciences, 366(1563), 389-401.

*To make this argument, I'd suggest focusing more heavily on learning curves in disgust and fear conditioning paradigms as these would point towards a biological preparedness in associative learning, which may rely on stable endophenotypes.*

**ACCEPTED and EXPLAINED**: Thank you for the idea, fast learning and resistance for the memory extinction t is one of the nice classical examples and it occurs in spiders (snakes). Actually there is very fast learning found in 9-month old infants that show increased attention to spiders previously cued with a fearful expression (Hoehl & Pauen, 2017). There are also classical studies which have paired pictures of spiders with aversive consequences like a mild electric shock. They have found generally that the fear which becomes conditioned to snake (or snake and spider pictures in such procedures is significantly more resistant to extinction than fear that is conditioned to fear-irrelevant stimuli such as pictures of flowers or houses etc. (for review of older papers see McNally, 1987; Davey 1992). Such findings suggest that, once acquired, fear of spiders and snakes may be relatively difficult to extinct.

There is also the review paper (Luck and Lipp 2016) reporting several papers using conditioning paradigm and comparing fear relevant (snakes and spiders) and fear irrelevant stimuli. They use special method, instructed extinction, which involves informing participants after the fear learning, that unconditional stimulus (electric shock) will no longer be present. This method facilitates extinction in fear irrelevant stimuli, however if the fear relevant stimuli were images of snakes and spiders, the fear was not sensitive to instructed extinction (Öhman et al., 1975; Hugdahl & Öhman, 1977; Hugdahl, 1978; Soares & Öhman, 1993; Lipp & Edwards, 2002). However, as the pictures of snakes as well as spiders were used in one group of fear relevant stimuli, we cannot determine the specific effect of images depicting spiders. However, more recent paper of Luck, Patterson and Lipp (2020) shows, that instructed extinction of conditioned fear is similarly effective for fear relevant biological stimuli (snakes and spiders) as well as for stimuli that represent modern threats.

Moreover, Ash et al. (2018) in their review summarized the recent evidence for fear conditioning of snakes and spiders. They found that increased resistance to extinction of conditioned responses to snake and spider pictures was found in 10 (31%) of the experiments, whereas 22 (69%) experiments did not support the hypothesis. They conclude that there is weak empirical support for this prediction of preparedness theory. For all this reason, the quick associative learning and resistance to extinction is rather complicated to interpret as a clean proof for biological preparedness to spider stimuli, but ability to learn associate spiders with fear face expression in very young age seems promising.

Åhs, F., Rosén, J., Kastrati, G., Fredrikson, M., Agren, T., & Lundström, J. N. (2018). Biological preparedness and resistance to extinction of skin conductance responses conditioned to fear relevant animal pictures: A systematic review. Neuroscience & Biobehavioral Reviews, 95, 430-437.

Davey, G. C. (1992). Classical conditioning and the acquisition of human fears and phobias: A review and synthesis of the literature. Advances in Behaviour Research and Therapy, 14(1), 29-66.

Hoehl, S., & Pauen, S. (2017). Do infants associate spiders and snakes with fearful facial expressions?. Evolution and Human Behavior, 38(3), 404-413.

Hugdahl, K. (1978). Electrodermal conditioning to potentially phobic stimuli: Effects of instructed extinction. Behaviour Research and Therapy, 16(5), 315-321.

Hugdahl, K., & Öhman, A. (1977). Effects of instruction on acquisition and extinction of electrodermal responses to fear-relevant stimuli. Journal of Experimental Psychology: Human Learning and Memory, 3(5), 608-618.

Lipp, O. V., & Edwards, M. S. (2002). Effect of instructed extinction on verbal and autonomic indices of Pavlovian learning with fear-relevant and fear-irrelevant conditional stimuli. Journal of Psychophysiology, 16(3), 176-186.

Luck, C. C., & Lipp, O. V. (2016). Instructed extinction in human fear conditioning: History, recent developments, and future directions. Australian Journal of Psychology, 68(3), 209-227

Luck, C. C., Patterson, R. R., & Lipp, O. V. (2020). “Prepared” fear or socio‐cultural learning? Fear conditioned to guns, snakes, and spiders is eliminated by instructed extinction in a within‐participant differential fear conditioning paradigm. Psychophysiology, 57(4), e13516.

McNally, R.J. (1987). Preparedness and phobias: A review. Psychological Bulletin, 101, 283-303.

Soares, J. J. F., & Öhman, A. (1993). Preattentive processing, preparedness and phobias: Effects of instruction on conditioned electrodermal responses to masked and non-masked fearrelevant. Behaviour Research and Therapy, 31(1), 87-95.

*Line 83: This line of evidence suggests that the fear and disgust response can be modified by non-evolutionary influences. Please elaborate further how this plays into your argument on the evolutionary perspective.*

**ACCEPTED and EXPLAIN:** This is not the argument for, but rather against. There are studies showing that spiders are detected faster, but there are also studies showing opposite result. Sorry, we try to reflect that situation is complicated and results on spiders concerning early attention are nonconclusive. We rewrite entire paragraph.

Evidence for quick detection shows that spiders are found faster among fear-irrelevant distractors (e.g. flowers) than flowers among spiders (Öhman et al., 2001). Spiders are also detected faster among cockroaches than vice versa (LoBue, 2010). They are better in quickly capturing our attention even when presented on the periphery of the visual field compared to a modern threatening stimulus (needle) or another fear-irrelevant animal (housefly) (New & German, 2015).

But, there are also studies, that we have mentioned in previous version of the manuscript, which show that sometimes spiders did not capture early visual attention.

LoBue, V. (2010). And along came a spider: An attentional bias for the detection of spiders in young children and adults. Journal of experimental child psychology, 107(1), 59-66.

New, J. J., & German, T. C. (2015). Spiders at the cocktail party: An ancestral threat that surmounts inattentional blindness. Evolution and Human Behavior, 36(3), 165-173.

Öhman, A., Flykt, A., & Esteves, F. (2001). Emotion drives attention: detecting the snake in the grass. Journal of experimental psychology: general, 130(3), 466.

*Line 85: To me, this sounds like the best argument for this perspective.*

**ACCEPTED:** Thank you for the suggestion. We agree that developmental studies brings the most important argument, we change the argumentation and we rewrite all the paragraph.

*Line 190: Table 1 - Please state how the pictures were randomized.*

**EXPLAINED:** The presentation of the picture during fear and disgust evaluation was randomized (this is going automatically in our testing website www.krasazvirat.cz), in addition to this randomization half of the respondents evaluated fear first, the other half evaluated pictures according the disgust first, then according the fear. We have two variants, either fear evaluation first or disgust evaluation first, thus the whole experiment was counterbalanced.

The clarifying sentence has been added to the Methods section: "The order in which were pictures presented was randomized separately for each respondent. The stimuli order for fear evaluation and for disgust evaluation were generated independently*,* 478 respondents rated the stimuli by perceived fear first and 490 by perceived disgust first.".

*Please include some kind of error indication (Standard error or standard deviation).*

**ACCEPTED:** The error indicators (Standard deviations) have been added to Table 1.

*Line 301: The Tables are missing.*

**ACCEPT and EXPLAINED:** All supplementary tables were provided. However, a technical issue may have occurred that caused the file to be saved incorrectly and could not be viewed. The Excel file (Supplementary) has been corrected. We hope it is now working correctly and all the tables are visible.

*Line 335: I'm not sure I understand why you used factor analyses for this purpose. The idea behind FA is to find latent, metric factors. FA will not be able to categorize latent classes. The factors extracted from this analysis would provide each participant with an estimation of 3(4) latent traits that had influence on their rating pattern. However, if these latent factors are defined as "hairy spiders", "general spiders", etc., no valid interpretation of the factor structure is possible. There is no interpretation like "participant 1 has a latent trait of 0.3 on the factor non-spider".
If you want to cluster participants with a latent structural equitation model, a latent class model instead of an exploratory factor analysis would make more sense to me.*

**ACCEPTED and EXPLANATION:** We do not attempt to categorize latent classes. We are searching for latent factors contributing to (fear- or disgust-) rating of the examined stimuli. Each participant has a combination of these factors predicting his/her fear (or disgust) ratings of the stimuli. The results of FA revealed that these factors are interpretable as considerable loadings correspond to interpretable groups of stimuli. But these groups are not a priori defined groups. However, you are right that the factor names ("Hairy spiders", "non-spiders", "general spiders", etc.) were not correctly chosen as the factors do not represent the category of the stimuli but rather the emotion elicited by the category of the stimuli. To make this clear, we renamed the factors to “Fear of hairy spiders”, “Disgust of general spiders” etc.

*Line 346: Tables are missing.*

**ACCEPTED:** See comment to Line 301.

*Line 381: Since I am not able to assess the FA in detail without the Tables and since I am also not sure about the use of FA in this context, I am excited to hear your comments on my suggestions stated above, before discussing this section further. However, I still want to make one remark: In many clinical contexts it looks like symptom severity heavily changes the pattern that participants score in questionnaires, possibly leading to different latent factor structures. As a result, by assessing latent classes of rated stimuli and classes of raters at the same time, analyses may run into some sort of circularity. Possibly, the classes will depend on who you ask (participant sub-groups/ extreme groups) and on the other hand class-scores or factor-scores used to assess differences between participants may hide or overestimate interindividual differences depending on the clusters/ scores you've extracted. Thus, I think that if you want to make the point of differences between groups of participants and on the other hand also want to assess latent classes, then you may want to think about separated stimulus- clustering for each sub-class of participants (i.e., phobics vs. individuals with low fear-scores). Differences between clusters across classes of participants may highlight differences in cognitive categorization Furthermore, I'd like to see some sort of discussion on why seperated analyses were conducted for fear and disgust, while the introduction makes the argument that the relation of fear and disgust is special for spiders in comparison to other stimuli. If so, why would you not want to take both dimensions into account at the same time/ in the same model? Basing structural equitation models on data that misses a relevant dimension may bias estimation.*

**ACCEPT and EXPLAINED:** To answer the first part of your comment about some participants possibly exhibiting a different pattern of rating, you are certainly right that this is often the cases. However, it is not in this study. In Table S14, you can find Spearman rank correlations of stimuli mean scores by the SPQ categories of respondents. Almost all of these correlations are significant, but even more importantly, many are very high. The correlation between the suspected phobic respondents and the high fear of spiders respondents (two categories with the highest SPQ scores) is 0.9756 for fear and 0.9595 for disgust. The correlation between the suspected phobic respondents (the highest SPQ category) and the moderate fear of spiders respondents (the middle SPQ category) is 0.8597 for fear and 0.7169 for disgust. The range of SPQ scores of these three categories together correspond to about 50 % of the sample of over 3,000 other Czech respondents who completed the SPQ questionnaire during a separate study. Therefore, the suspected phobic respondents should not be considered an extreme group since their pattern of rating is very similar to that of about a half of the general population.

Nevertheless, the solution you suggest is also valid and we indeed tried this before. However, by the naked eye, we found no significant differences. Further, we found that both suspected phobic respondents and extremely low fear of spiders respondents tend to use a very limited scores for at least half of the stimuli (6 and 7 in the first case, 1, 2 and 3 in the second). The low range and diversity of rating reduces the credibility of factor analysis in general, so this is the second reason we chose to include only the analysis for all the respondents together.

As for the second part of your comment, we performed the factor analysis including scores from both fear and disgust and extracted seven factors (as suggested by parallel analysis). With some degree of simplification, the first factor corresponds to the fear of gracile spiders and other chelicerates, the second to fear and disgust of myriapods, earwig and woodlouse, the third to fear and disgust of hairy robust spiders and gracile but dark spiders (the later also contribute to the first or fourth factor), the fourth factor to the disgust of gracile spiders and other chelicerates, the fifth factor to the fear and disgust of crabs and the water measurer, the sixth factor to the fear and disgust of the tick and mite, and finally the seventh factor cannot be interpreted. The results can be found in the Table R1 below. As you can notice, the factors usually consist of certain stimuli’s both fear and disgust rating. There is only one exception – the gracile spiders and other chelicerates – that contributes to the first factor (only) by their fear scores and to the fourth factor (only) by their disgust scores. But even in this case, the stimuli in the first and fourth factor correspond remarkably well. Based on this, we concluded that there is no “interaction” between the stimuli identity and the rated emotion meaning there are no groups of stimuli that unevenly contribute to one factor by their fear scores but to another factor by their disgust scores. This in combination with pragmatic reasons (e.g., simpler presentation to the reader) lead the decision to present two separate factor analyses. Further, while not a direct aim of the study, it allowed us to discus which emotion is more probably at the background of the origins of negative emotions elicited by spiders.

We added a short discussion on why we chose to primarily perform two separate factor analyses and base next computations and following discussion on their results. However, it still is a very good point and we included new version of the S7 Table in the Supplement as an alternative results.

*Line 405: I can't tell if this is significant or not without error indicators.*

**ACCEPTED:** The error indicators (Standard deviations) have been added to Table 1. You can also see formal comparison of the means in Tables S3 and S4 (which are now hopefully visible).

*Line 425: Were stimuli not randomized?*

**EXPLAINED:** Yes, they were, however multiple spider stimuli could still overshadow a single tick even in a random presentation order design. As there was only one tick, statistically, it would be presented to the average respondent as the 24^th^ stimuli (in the middle of the presentation). As there were 25 spider stimuli, by chance, a half of them should be presented in the first half of the presentation, and the other half in the second part of the presentation. Therefore, the average respondent would be presented with 12 or 13 spider stimuli before seeing the tick.

*Line 519: Is this effect present after correction for SPQ scores? if not, this interpretation would be oversimplyfied.*

**EXPLAINED:** This interpretation is based on the results of RDA where the SPQ score was included. Therefore, the effect remains even after the correction for SPQ score.

**Reviewer2:** Background and Evaluation:
This study examines the sensation of fear and disgust for spider stimuli compared to other arthropods. In an online survey, participants evaluated a number of stimuli (spiders, non-spider chelicerates, and other arthropods) on a 7-point scale regarding the level of fear and disgust they experience. They found out that spiders as stimuli are evaluated higher on fear and disgust than other arthropods. In addition, a vast majority of spiders scored higher in fear than disgust, and vice versa for other chelicerates and arthropods. Results were similar among almost all different groups of responders, highlighting the evolutionary aspect of fear of spiders.
My overall evaluation is positive. Strength points include an interesting topic that is relevant to anxiety research. As a reader, the article provides a very logical explanation for the theoretical background (evolutionary significance), the findings and its implications. Additionally, the study has a big sample size. My suggestion is to improve the paper in regards of the theoretical background (SPQ vs FSQ and “cognitive category”) . My detailed comments are outlined below. Please note that my comments are aimed to further improving the manuscript, not to criticize the author’s work.

*Page 3, line 48: The ‘fear of spider questionnaire’ is FSQ. The SPQ is called ‘spider phobia questionnaire’ (Klorman et al., 1974). Even though the questionnaires are similar in structure and content, they are different questionnaires (Szymanski & O’Donohue, 1995).*

**ACCEPTED:** The name of the questionnaire has been corrected. The Spider Phobia Questionnaire (Klorman et al., 1974) were used in this study, but we accidentally typed the wrong name into the text.

*Page 4, line 6: “Related idea..” sentence is difficult to read.*

**ACCEPTED:** The sentence has been rewritten: " Related idea posits that, in human ancestors,

disgust has increased avoidance of pathogens, parasites and possible sources of contamination."

*Page 6, line 122: Please further explain “Do spiders form a single distinct cognitive category?”. I suggest either rewrite this question a little or provide more background as it is difficult to understand what “cognitive” means here. Some explanation is provided in the discussion section (page 20, lines: 440-448) and theoretical background (page 4, lines 84-85), however, I suggest to further elaborate “cognitive” category: One could interpret this as different emotional categories, and this would be very similar to research question #1.*

**ACCEPTED and EXPLAINED**: Thank you for your comment. We are aware that there are several theories that define what a cognitive category is.

In this work, we focused on the perception of negative emotions from selected groups of invertebrates, as well as on what humans consider (categorize as a) a spider. During categorization process the categories are very often formed also on the basis of existing similarity among the stimuli. Thus providing enough variability of stimuli with certain level of similarity as well as difference is very important. This issue is very difficult achive, if we want to work with natural stimuli, and cover important morphotypes of chelicerates and other similar invertebrates at the same time.

**Unfortunately, most of the previous works dealing with spiders as an animals, from which phobias most often arise, does not take into account the great morphological diversity of both spiders and their relatives from the Chelicerata group. So, our first question is about how respondents evaluate according the emotions true spiders (as a taxonomic group) and other taxa.**

**The second question is about how respondence perceive spider category (in this meaning the term perceptual category is used, here we accented the term cognitive category, to accent existence of category inside the human mind). Here we try to find how wide is the cognitive category, without any taxonomic predictions (we only discuss the results in light of existing taxonomy). We were interested in whether people consider as the spiders only those species that truelly belong here, or extend this category to other spider-like species, or, conversely, do not perceive some species as a spider.**

**An example of such spontaneous categorization of animals according to emotional and perceptual criteria can be found in the work on reptiles (Janovcová et al. 2019). Here, humans distinguished reptiles on the basis of emotional evaluation and some important morphological traits into two cognitive categories. The stimuli were categorized according to the presence of limbs, although they taxonomically belong to different groups of reptiles, namely snakes, legless lizards and amphisbaens vs. turtles, crocodiles and lizards with limbs. Moreover, inside each of these two categories were gradient according the level of perceived fear or disgust.**

**In this study, for the both questions, we can explore the morphological traits of the original stimuli and how they help to form the category.**

**However, you are correct, that the second question how wide is the “spider ” inner category in our mind, is more important for our line of argumentation. As the term category have different meanings (primary category, perceptual category, emotional category) coming from different frames of behavioural sciences.**

**How we calculate the results from emotional evaluation according fear and disgust to answer this two questions?**

In the first question, we are interested if taxon of spiders are rated as low, middle, or high fear-eliciting and as low, middle, or high disgust-eliciting. We are also interested in whether spiders score high or low in both scales, or higher on one scale but lower on the other. In both cases, we compare their rating to rating of other stimuli, especially other species of chelicerates and we test whether they are similar or different.

**In the second question, we focus on the rating pattern of the respondents. We no longer ask whether a particular respondent rates spiders high or low in fear but whether he/she rates all spiders similarly (gives them a similar score). If a pair of animals always receives two same scores, then it is reasonable to conclude that respondents perceive them as two very similar animals even if some respondents scored them very low and some very high. Then, such animals are a part of one cognitive category. In our study, we try to delineate these cognitive categories by analysing ratings on two scales – fear and disgust – of which both are emotional. However, the scale is inconsequential in this second question (but not the first one) as we aim to access cognitive categories which should be stable across many contexts and many respondents.**

The first and second question has been rewritten: " (1) Is position of taxonomically defined spiders on fear and disgust scales distinctive compared to that of other chelicerates and arthropods? (2) Do spiders form a single distinct cognitive category or more species of invertebtrates are perceived as a "spider "?"

We also added part about categorization and the definition of cognitive category to the Introduction section: “Emotions can direct automatic attention to emotionally salient stimuli (Carretié 2014) like snakes and spiders and sometimes precede even conscious perception (Barrett and Simmons 2015). However, perception as a cognitive process of transformation of proximal stimulus into a percept (the accessible, subjective experience that is connected with activation of a certain category in the mind; Brosch, Pourtois, Sander 2010) modulates further late attention towards evolutionary relevant threatening stimuli (Grassini et al. 2019). Michalovski et al. (2009) studied temporal dynamics of visual attention to the spiders and other pleasant and unpleasant stimuli using ERPs (event related potentials). They found, that the spiders are processed preferentially in later stages of perceptual and evaluative processing, especially in spider phobics. Late cognitive stimulus evaluation, like its proper categorization, is thus important when we are confronted with classes of stimuli that have (or had in our evolutionary past) more direct relevance for our well-being and survival than others. We can expect that extremely relevant stimuli are categorized into special emotional categories in human mind, which may differ from other categories (Brosch, Pourtois, Sander 2010), and they are preferentially processed in the brain (Brosch, Sander, Pourtois, & Scherer, 2008; Pool, Brosch, et al., 2016). Forming the stimulus category in the mind is thus a cognitive process when people group certain objects or concepts as equivalent or analogous reducing the information complexity, but they acquire set of information thanks to association of the object with a certain category (Harnad 2017). Proper categorization of the potentially life-threatening stimuli may still direct our late attention on the one hand, but may allow for effective regulation of the impact of negative emotions like fear or disgust on the other one (Park and Thayer 2014).Categorization of emotional stimuli as a cognitive process assumes the existence of categories based on the everyday experience or evolutionary past in some cases. If people with very different experiences form similar emotional categories containing life-threatening animal stimuli like scorpions as well as harmless spiders, it may indicate the existence of a pre-existing general category for these incentives in human mind, which may be generalized to a wider group of animal species. This argument supports the hypothetical existence of evolutionarily rooted negative emotions of specific animal stimuli, similarly to a more frequently used argument of the cross-cultural agreement in emotional evaluation of these stimuli (Prokop et al. 2010).

In conclusion, we hypothesise that specific animal stimuli like spiders (and may-be other more dangerous arthropods) may form the category inside the human mind on the basis of shared morphological features which we can perceived via our sensory system and they interact with emotional processing during its perception. The aspect that we accented here is, that people forming the category inside the human mind along with emotional evaluation, which is the cognitive process. Thus, we referred this category inside the human mind as the cognitive category further in the text . “

Barrett, L. F., & Simmons, W. K. (2015). Interoceptive predictions in the brain. Nature reviews neuroscience, 16(7), 419-429.

Brosch, T., Pourtois, G., & Sander, D. (2010). The perception and categorisation of emotional stimuli: A review. Cognition and emotion, 24(3), 377-400.

Brosch, T., Sander, D., Pourtois, G., & Scherer, K. R. (2008). Beyond fear: Rapid spatial orienting toward positive emotional stimuli. Psychological science, 19(4), 362-370.

Carretié, L. (2014). Exogenous (automatic) attention to emotional stimuli: a review. Cognitive, Affective, & Behavioral Neuroscience, 14(4), 1228-1258.

Grassini, S., Railo, H., Valli, K., Revonsuo, A., & Koivisto, M. (2019). Visual features and perceptual context modulate attention towards evolutionarily relevant threatening stimuli: Electrophysiological evidence. Emotion, 19(2), 348.

Harnad, S. (2017). To cognize is to categorize: cognition is categorization. In Handbook of categorization in cognitive science (pp. 21-54). Elsevier.

Michalowski, J. M., Melzig, C. A., Weike, A. I., Stockburger, J., Schupp, H. T., & Hamm, A. O. (2009). Brain dynamics in spider-phobic individuals exposed to phobia-relevant and other emotional stimuli. Emotion, 9(3), 306.

Park G, Thayer JF. From the heart to the mind: cardiac vagal tone modulates top-down and bottom-up visual perception and attention to emotional stimuli. Frontiers in psychology 2014; 5, 278.

Pool, E., Brosch, T., Delplanque, S., & Sander, D. (2016). Attentional bias for positive emotional stimuli: a meta-analytic investigation. Psychological bulletin, 142(1), 79.

Prokop, P., Tolarovičová, A., Camerik, A. M., & Peterková, V. (2010). High school students’ attitudes towards spiders: A cross‐cultural comparison. International Journal of Science Education, 32(12), 1665-1688.
